# Hedonic hotspot in rat olfactory tubercle: map for mu-opioid, orexin, and muscimol enhancement of sucrose ‘liking’

**DOI:** 10.1038/s41386-026-02374-6

**Published:** 2026-03-03

**Authors:** Koshi Murata, Kent C. Berridge

**Affiliations:** 1 Department of Psychology, University of Michigan, Ann Arbor, MI, USA.; 2 Division of Brain Structure and Function, Faculty of Medical Sciences, University of Fukui, Fukui, Japan.; 3 Life Science Innovation Center, University of Fukui, Fukui, Japan.

## Abstract

Pleasure plays a crucial role in positive reinforcement and motivation. Brain regions able to amplify positive hedonic reactions to sweetness, known as ‘hedonic hotspots’, are distributed within the mesocorticolimbic reward systems. The olfactory tubercle (OT), a part of the ventral striatum that receives olfactory input, contains distinct functional domains: the anteromedial domain mediates approach motivation toward odors associated with food, whereas the lateral domain mediates avoidance motivation away from odors associated with danger. However, it has remained unclear whether the OT modulates hedonic reactions to pleasant sensations. In this study, we made pharmacological microinjections in OT of rats to examine whether these OT subregions can modulate hedonic reactions, as assessed by the taste reactivity test. Sweet oral infusions of sucrose solution were delivered into the mouth via an intraoral cannula, and the rats’ orofacial and somatic hedonic reactions were recorded and analyzed. We compared three pharmacological agents: mu-opioid receptor agonist DAMGO, orexin-A peptide, and GABA_A_ receptor agonist muscimol. Microinjection of any of these drugs into the anteromedial OT subregion enhanced hedonic ‘liking’ reactions to sucrose. Furthermore, DAMGO injection into the anteromedial OT subregion recruited distant Fos expression in other ‘hedonic hotspots’, including in the caudal ventral pallidum and the rostromedial orbitofrontal cortex. By contrast, the same microinjections into the anterolateral OT subregion failed to enhance ‘liking’ reactions and, DAMGO oppositely increased aversive ‘disgust’ reactions. These findings suggest that the anteromedial OT contains a ‘hedonic hotspot’, whereas the anterolateral OT may contain a suppressive opioid ‘hedonic coldspot’. Thus, OT subregions may help causally modulate hedonic reactions to sweetness and flavor perception.

## INTRODUCTION

Hedonic reactions to pleasant sensations play roles in motivating adaptive behaviors of animals and humans essential for survival. To elucidate neurobiological mechanisms underlying pleasure, it is essential to objectively measure affective ‘liking’ reactions elicited by the hedonic impact of pleasant stimuli such as sensory rewards. Affective neuroscience studies have often used the taste reactivity test, which quantifies distinct orofacial expressions evoked by pleasant versus unpleasant tastes, to measure ‘liking’ reactions to sweet tastes and ‘disgust’ reactions to bitter tastes [1, 2]. Rodents, non-human primates, and human infants share many of these affective facial reactions.

Pharmacological microinjections and optogenetic manipulations of neural systems have revealed hedonic mechanisms by mapping the ability of neurobiological modulations to causally change hedonic orofacial ‘liking’ reactions to tastes [3, 4]. In particular, this mapping has identified multiple hedonic hotspots across the brain, which are small restricted subregions within mesocorticolimbic structures that are especially able to enhance ‘liking’ reactions to sweetness in response to local neurobiological manipulations. Identified hedonic hotspots include the rostrodorsal quadrant of medial shell of nucleus accumbens (NAc), the caudolateral half of ventral pallidum (VP), a rostromedial portion of the orbitofrontal cortex (OFC), a far posterior zone of the insula cortex, and the parabrachial nucleus of the brainstem pons [5-9]. These hedonic hotspots that generate ‘liking’ are embedded within larger mesocorticolimbic circuitry that can generate incentive salience or ‘wanting’, suggesting a close interconnection between ‘liking’ and ‘wanting’ mechanisms in reward [10].

The olfactory tubercle (OT), despite its olfactory name, is actually a rostroventral extension of the ventral striatum, sharing several neurobiological features and being anatomically continuous with the NAc [11-13]. For example, both the OT and NAc contain GABAergic principal neurons known as medium spiny neurons (MSNs), whose primary axonal target is the VP. Both OT and NAc MSNs express dopamine receptors, mostly expressing either D1 dopamine receptors and the opioid peptide dynorphin, or D2 dopamine receptors and the opioid peptide enkephalin [14, 15]. Therefore, the OT has also been referred to as the “olfactory striatum” or “tubular striatum” [16, 17].

The OT also mediates reward and motivation functions similarly to NAc. For example, rats self-administer intracranial microinjections of addictive drugs in OT, particularly within its anteromedial subregion (OTam) [18-20]. Involvement of OTam in odor-guided appetitive behaviors has been demonstrated by mapping Fos activation elicited during attraction to an odor cue paired with sugar-reward [21]. Further, mesolimbic dopamine inputs from the ventral tegmental area (VTA) to the medial OT mediate the acquisition and execution of odor preference [22]. By contrast, the anterolateral OT (OTal) appears to participate in aversive behavior, as indicated by Fos activation in OTal during aversion to an odor cue paired with foot-shock [21]. Similarly, the caudal subregion of NAc medial shell has been implicated in several aversive behaviors in rats [23, 24]. These structural and functional parallels between the OT and NAc, together with evidence that the OT contributes to motivational incentive salience or reward ‘wanting’, raise the possibility that OT might also contain hedonic hotspots capable of enhancing ‘liking’ reactions, similarly to the NAc. However, to our knowledge, no studies have directly tested for hedonic hotspots within the OT.

Here, we used pharmacological microinjections to investigate whether the OTam, previously implicated in reward motivation, contains a hedonic hotspot capable of amplifying ‘liking’ reactions to sucrose taste. Previous studies of the NAc hedonic hotspot of the rostrodorsal medial shell showed that sucrose ‘liking’ reactions were enhanced by local microinjections of any of: the mu opioid receptor agonist DAMGO, orexin-A peptide, or the GABA_A_ receptor agonist muscimol [7, 24, 25]. Therefore, we tested whether microinjections of any of these three drugs into the anteromedial or anterolateral OT subregions would alter affective ‘liking’ reactions to sucrose taste in rats. Those anatomically distinct OT subregions play different roles in physiological and behavioral responses, and their functional organization is distributed along the medio-lateral axis [26]. Our results indicate that microinjections of DAMGO, orexin, or muscimol into the OTam enhanced hedonic ‘liking’ reactions elicited by oral sucrose infusions over control vehicle levels measured in the same rats, identifying the OTam as an OT hedonic hotspot. In contrast, the same microinjections in the OTal did not enhance ‘liking’ and, in some cases, produced oppositely valenced aversive effects. Furthermore, Fos mapping revealed that DAMGO microinjection into the OTam induced concurrent activation in other hedonic hotspots (the caudal VP and rostromedial OFC), suggesting that the OTam functions as a part of a distributed hedonic hotspot network.

## MATERIALS AND METHODS

### Animals

Female (*n* = 20) and male (*n* = 18) Sprague–Dawley rats (230–460 g) were used. Of these, 14 females and 12 males were assigned to taste reactivity experiments, and 6 females and 6 males to Fos mapping. Rats were housed 2–4 per cage at 21 °C under a reverse 12-h light/dark cycle with ad libitum food and water. All procedures were approved by the University of Michigan Institutional Animal Care and Use Committee.

### Surgery

Rats were anesthetized with isoflurane (4–5% induction, 1–2% maintenance) and placed in a stereotaxic apparatus (incisor bar 5.5 mm below intra-aural zero). Bilateral guide cannulas were implanted into either the anteromedial (OTam) or anterolateral (OTal) olfactory tubercle, targeting 2 mm above the dense cell layer. For the OTam-targeted group, skull holes were bilaterally drilled at +2.3 mm anterior and ±3.0 mm lateral to bregma, and the guide cannulas were inserted at a 15° angle toward the midline to a depth of 8.0 mm from the skull surface. For the OTal-targeted group, skull holes were bilaterally drilled at +1.8 mm anterior and ±2.8 mm lateral to bregma, and the guide cannulas were vertically inserted (0° angle) to a depth of 8.0 mm. For taste reactivity experiments, bilateral intraoral cannulas (PE-100 tubing) were implanted to permit infusion of sucrose solutions [27]. Rats received postoperative analgesia and antibiotics and recovered for at least 7 days before testing.

### Drug microinjections

Bilateral microinjections (0.2 μL/side, 1 min) were made using injectors extending 2 mm beyond the guide cannulas. Drugs included the mu-opioid receptor agonist DAMGO (50 ng/side), orexin-A peptide (500 pmol/side), GABAA receptor agonist muscimol (75 ng/side), and ACSF vehicle alone. Each rat received only one treatment per day, and the order was counterbalanced. Drug doses were selected based on previous studies [24, 25, 28].

### Taste reactivity tests

The taste reactivity test [2, 27, 29] was used to measure affective orofacial reactions elicited by intraoral infusion of a sucrose solution (1% w/v). A 1-mL volume of sucrose solution was delivered into the mouth via oral cannula over a 1-min period. Infusions were administered 25 min after microinjections, approximately when peak pharmacological effects could be expected [24, 25, 28]. Orofacial and somatic reactions were videorecorded (30 fps) with ventral mirror views. Taste reactivity components that strongly reflect hedonic ‘liking’ include lateral tongue protrusions, paw licks, and rhythmic midline tongue protrusions (Berridge, 2000) (Fig. S1). Rhythmic tongue protrusions are typically elicited reflexively by sweet taste infusions in bouts lasting up to several seconds. Very highly palatable sweet tastes can also elicit additional longer bouts of lateral tongue protrusions, and even paw licking. In paw licking, the taste-elicited tongue protrusions are directed toward the forepaws, which are held up to the mouth, as if the orally infused solution were being licked off the forepaws, and paw licking can persist continuously for several seconds to up to half a minute during a 1-min oral infusion. Both hedonic ‘liking’ and aversive ‘disgust’ (gapes, head shakes, face washes, forelimb flails, and chin rubs, Fig. S2) responses were scored offline frame-by-frame using standardized bin-based criteria to ensure equal weighting of frequent and rare behaviors. Totals of hedonic and aversive reactions were computed for each drug and control condition. In the present study, “enhancement” of hedonic responses refers specifically to an increase in the magnitude or frequency of taste-evoked positive orofacial reactions elicited during sucrose infusion, relative to control conditions. The term does not imply generation of hedonic reactions in the absence of sensory stimulation.

### Histology for cannula placement and mapping of behavioral effects

After testing, rats were microinjected with fluorescent tracer (Red RetroBeads) for anatomical verification, then perfused with 4% paraformaldehyde. Brains were sectioned (50 µm) and examined under fluorescence microscopy. Cannula placements were plotted on atlas templates [30], and functional maps were constructed showing anatomical distributions of hedonic enhancement and suppression relative to vehicle baselines.

### Fos analysis

A separate cohort received DAMGO or vehicle injections in the OTam under identical conditions. Brains were collected 85 min post-injection and processed for Fos immunohistochemistry (rabbit anti-c-Fos (MERCK/Sigma-Aldrich ABE457) and Cy3-conjugated secondary). Local ‘Fos plumes’ around injection sites were quantified as zones of elevated Fos cell density relative to vehicle controls (Fig. S3), and the average plume radius was used to scale symbol sizes on functional maps (Fig. 5C remapped from Fig. 2A). Fos cell density was also quantified in distant mesocorticolimbic structures (OFC, prelimbic cortex, infralimbic cortex, insula, anterior cingulate cortex (ACC), NAc medial shell and core, VP, central amygdala (CeA), basolateral amygdala (BLA), medial amygdala (MeA), lateral hypothalamus (LH), perifornical area of hypothalamus (PFA), arcuate nucleus of hypothalamus, VTA, and paraventricular thalamus) to assess distributed network activation (Fig. S4), guided by a template derived from a corresponding brain atlas to ensure consistent placement [31].

### Statistics

**Fig. 1:**
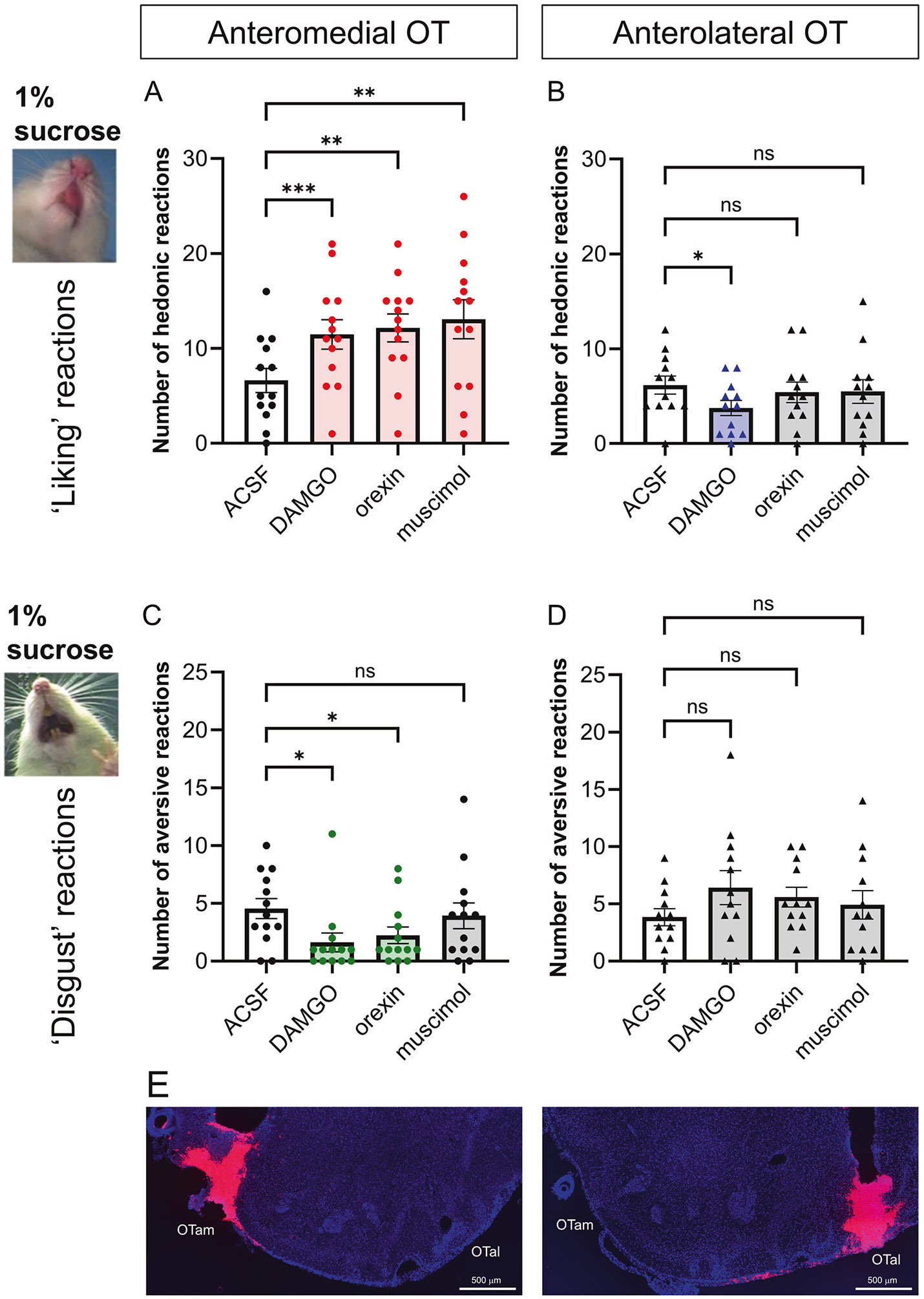
Hedonic ‘liking’ enhancement by microinjections of DAMGO, orexin, and muscimol within the OTam. Quantification of hedonic ‘liking’ (**A**, **B**) and aversive ‘disgust’ (**C**, **D**) reactions to sucrose taste following vehicle (ACSF), DAMGO, orexin, or muscimol microinjections into the OTam (**A**, **C**) and (**B**, **D**). **E** Representative cannula placements within the OTam (left) and OTal (right) identified by fluorescence tracer (red) and DAPI staining (blue). *n* = 13 rats for (**A**, **C**); *n* = 12 rats for (**B**, **D**). Data are shown as mean ± SEM. *p < 0.05, **p < 0.01 by Dunnett’s test.

**Fig. 2:**
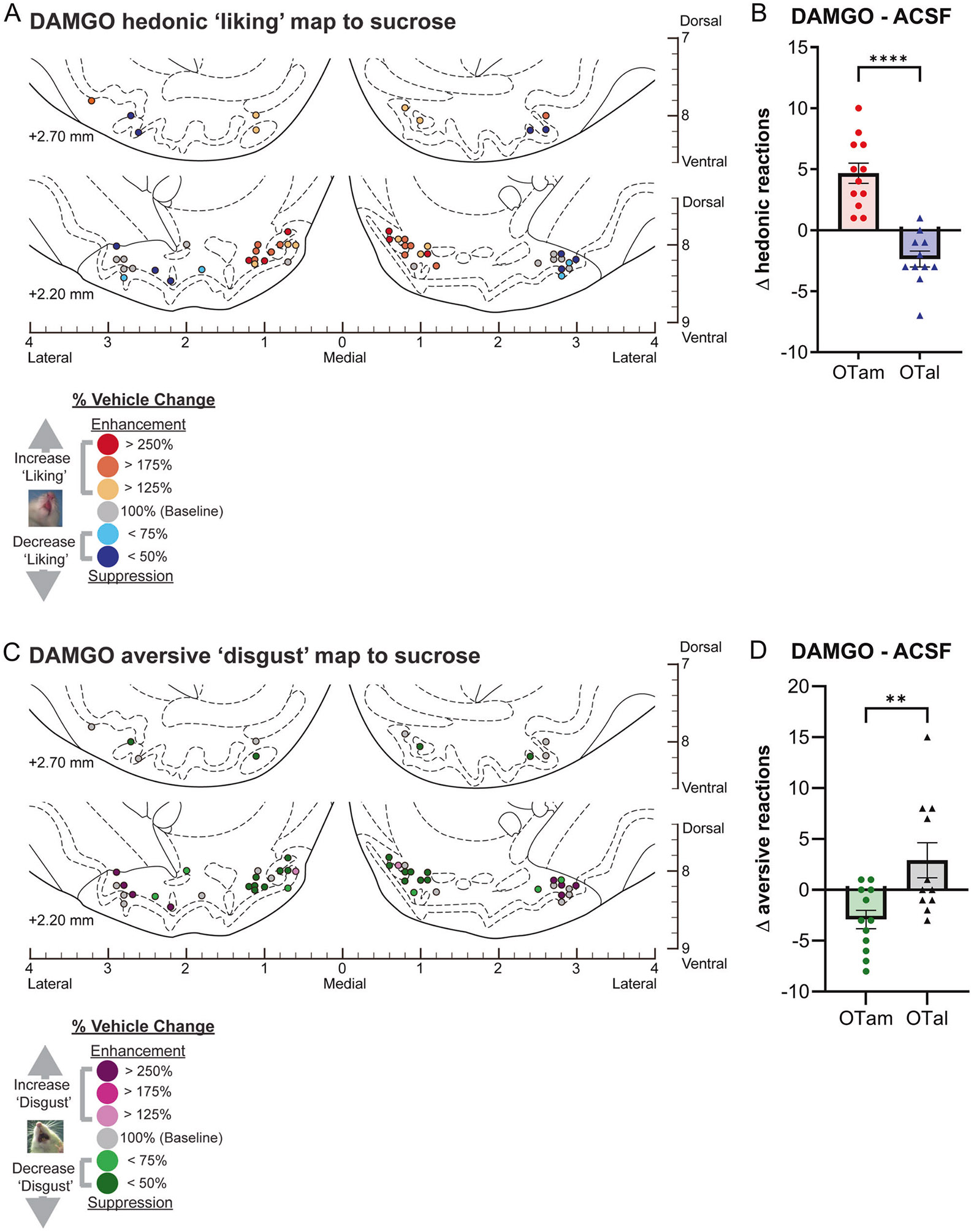
Causation OT maps of hedonic “liking” enhancement by DAMGO. **A** Localization of hedonic function map shows how DAMGO microinjections altered ‘liking’ reactions to intraoral sucrose infusions at each individual OT site. Each symbol indicates the putative cannula tip location for an individual rat plotted on a coronal brain atlas [30]. Symbol color represents within-subject behavioral changes in hedonic reactions induced by DAMGO, expressed as percent change from vehicle ACSF microinjection control levels in the same rat (‘liking’ enhancement: red-yellow; ‘liking’ suppression: blue). **B** DAMGO microinjections differentially alter hedonic ‘liking’ reactions depending on the anatomical subregion of the OT. DAMGO microinjections in the OTam enhanced hedonic ‘liking’ reactions, while those in the OTal suppressed them. **C** Localization of aversive function map shows how DAMGO microinjections altered ‘disgust’ reactions to intraoral sucrose infusions at each individual OT site. Each symbol indicates the putative cannula tip location for an individual rat plotted on a coronal brain atlas [30]. Symbol color represents within-subject behavioral changes in hedonic reactions induced by DAMGO, expressed as percent change from vehicle ACSF microinjection control levels in the same rat (‘disgust’ enhancement: magenta; ‘disgust’ suppression: green). **D** DAMGO microinjections differentially alter aversive ‘disgust’ reactions depending on the anatomical subregion of the OT. DAMGO microinjections in the OTam suppressed aversive ‘disgust’ reactions, whereas those in the OTal showed no significant effect. *n* = 13 rats for (**A**, **B**); *n* = 12 rats for (**C**, **D**). Data are shown as mean ± SEM. ****p < 0.0001; **p < 0.01 by Weltch’s *t* test.

**Fig. 3:**
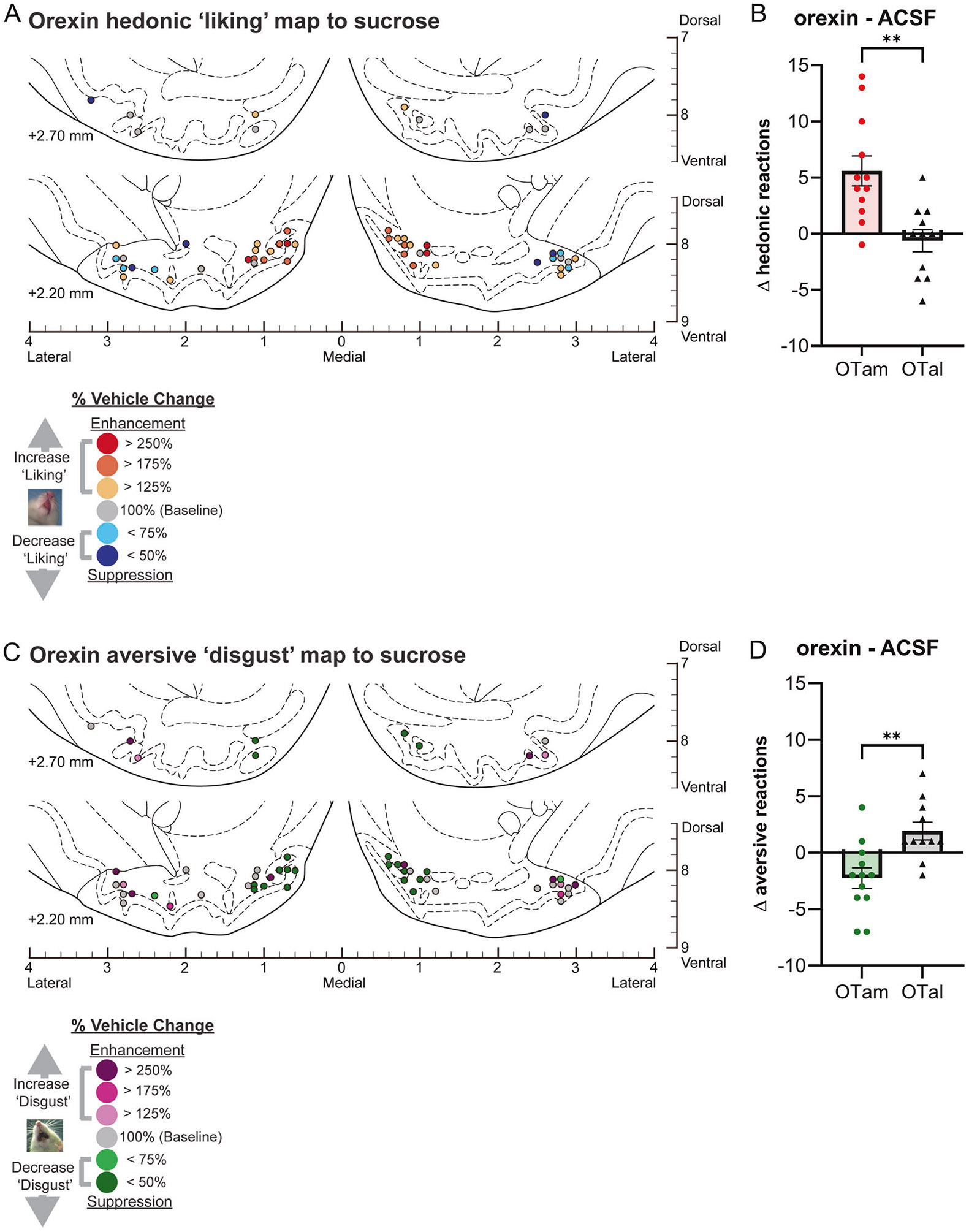
Causation OT maps of “liking” enhancement by orexin. **A** Localization of hedonic function across OT sites showing how orexin microinjections altered ‘liking’ reactions to intraoral sucrose infusions at each individual site. Each symbol represents the putative cannula tip location for an individual rat plotted on a coronal brain atlas [30]. Symbol color indicates within-subject behavioral changes in hedonic reactions induced by orexin, expressed as percent change from vehicle (ACSF) control levels in the same rat (‘liking’ enhancement: red–yellow; ‘liking’ suppression: blue). **B** Orexin microinjections differentially altered hedonic ‘liking’ reactions depending on the anatomical subregion of the OT. Orexin microinjections in the OTam enhanced hedonic ‘liking’ reactions, whereas those in the OTal produced no significant change. **C** Localization of aversive function across OT sites showing how orexin microinjections altered ‘disgust’ reactions to intraoral sucrose infusions at each individual site. Each symbol represents the putative cannula tip location for an individual rat plotted on a coronal brain atlas [30]. Symbol color indicates within-subject behavioral changes in aversive reactions induced by orexin, expressed as percent change from vehicle (ACSF) control levels in the same rat (‘disgust’ enhancement: magenta; ‘disgust’ suppression: green). **D** Orexin microinjections differentially altered aversive ‘disgust’ reactions depending on the anatomical subregion of the OT. Orexin microinjections in the OTam suppressed aversive ‘disgust’ reactions, whereas those in the OTal produced no significant effect. *n* = 13 rats for (**A**, **B**); *n* = 12 rats for (**C**, **D**). Data are shown as mean ± SEM. **p < 0.01 by Welch’s *t* test.

**Fig. 4:**
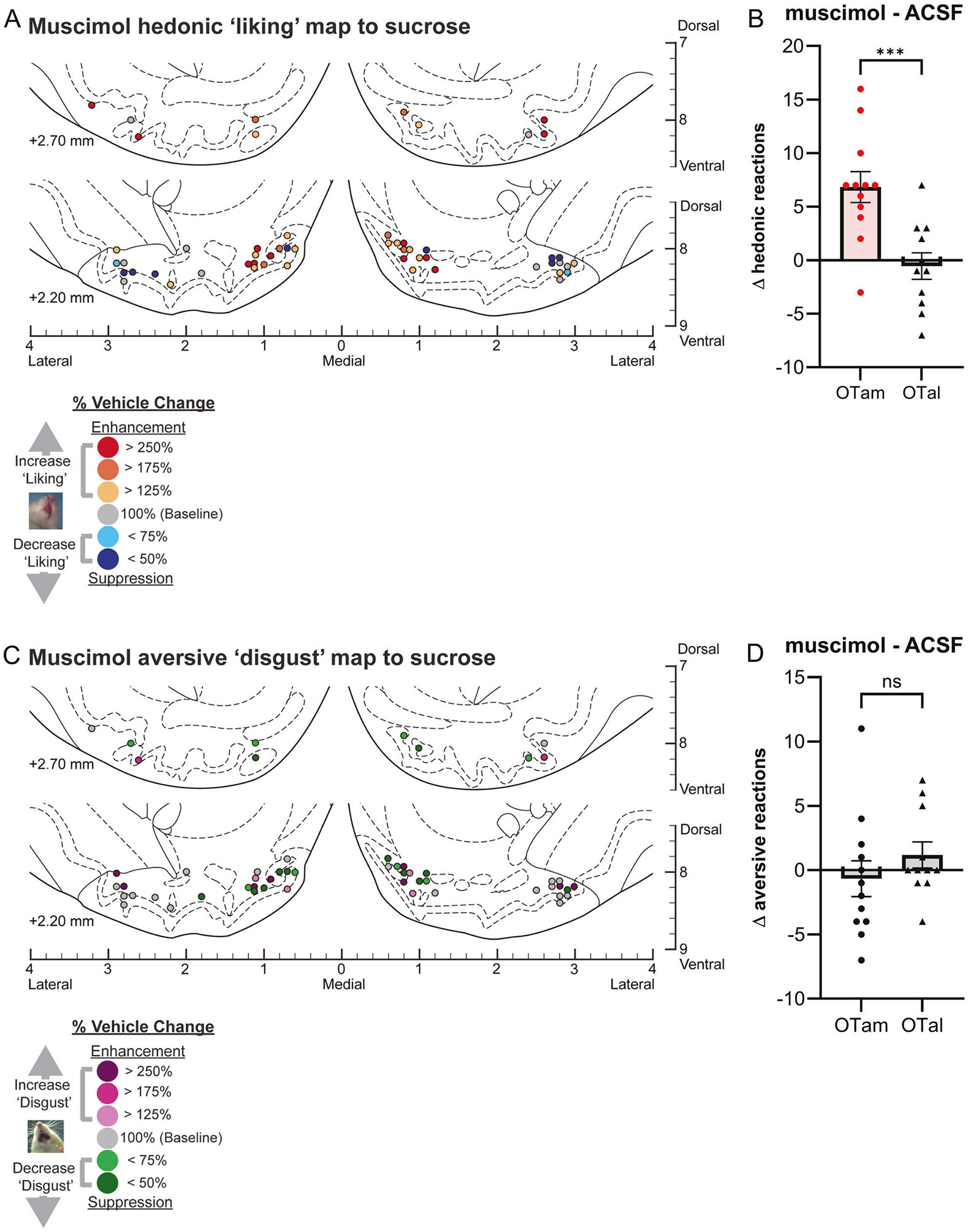
Causation OT maps of “liking” enhancement by muscimol. **A** Localization of hedonic function map shows how muscimol microinjections altered ‘liking’ reactions to intraoral sucrose infusions at each individual OT site. Each symbol indicates the putative cannula tip location for an individual rat plotted on a coronal brain atlas [30]. Symbol color represents within-subject behavioral changes in hedonic reactions induced by muscimol, expressed as percent change from vehicle ACSF microinjection control levels in the same rat (‘liking’ enhancement: red-yellow; ‘liking’ suppression: blue). **B** Muscimol microinjections differentially alter hedonic ‘liking’ reactions depending on the anatomical subregion of the OT. Muscimol microinjections in the OTam enhanced hedonic ‘liking’ reactions, whereas those in the OTal produced no significant change. **C** Localization of aversive function map shows how muscimol microinjections altered ‘disgust’ reactions to intraoral sucrose infusions at each individual OT site. Each symbol indicates the putative cannula tip location for an individual rat plotted on a coronal brain atlas [30]. Symbol color represents within-subject behavioral changes in hedonic reactions induced by muscimol, expressed as percent change from vehicle ACSF microinjection control levels in the same rat (‘disgust’ enhancement: magenta; ‘disgust’ suppression: green). **D** Muscimol microinjections did not significantly alter aversive ‘disgust’ reactions in either the OTam or OTal subregion. *n* = 13 rats for (**A**, **B**); *n* = 12 rats for (**C**, **D**). Data are shown as mean ± SEM. ***p < 0.0001 by Weltch’s *t* test.

**Fig. 5:**
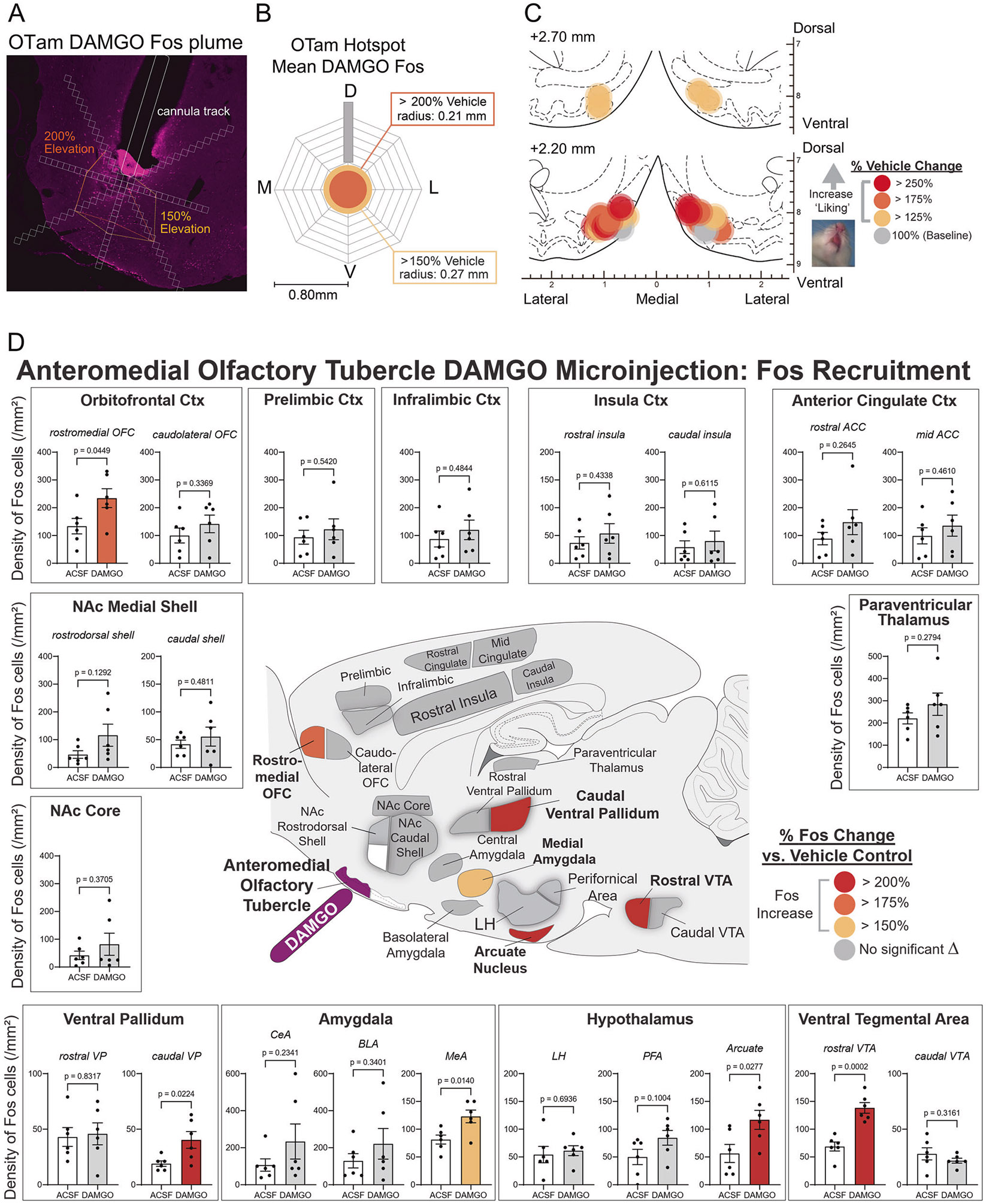
Fos plume and mapping of DAMGO microinjection in the OT anteromedial hotspot. **A** Representative photomicrograph showing local Fos plumes surrounding the cannula tip within the OTam hotspot. **B** Average size of Fos plumes. DAMGO, *n* = 6 rats; vehicle, *n* = 6 rats. D dorsal, M medial, V ventral, L lateral. **C** Remapping of hedonic function localization (from Fig. 2A) within the OTam revealed by DAMGO microinjections, with symbol size reflecting the average radius of Fos plumes. Each symbol represents the putative cannula tip location for an individual rat plotted on a coronal brain atlas [30]. Symbol color indicates within-subject behavioral changes in hedonic reactions induced by DAMGO, expressed as percent change from vehicle (ACSF) control levels in the same rat (‘liking’ enhancement: red–yellow). **D** Distant Fos recruitment following DAMGO microinjection into the OTam hotspot. Brain map illustrates increased Fos cell density in mesocorticolimbic structures recruited for hedonic enhancement. Colors denote significant percent Fos elevation relative to vehicle control rats. Significant Fos increases were observed in other hedonic hotspots, including the caudal VP and rostromedial OFC. Fos elevation was also recruited in other limbic regions, such as rostral VTA, arcuate nucleus of the hypothalamus, and medial amygdala. DAMGO, *n* = 6 rats; vehicle, *n* = 6 rats. Bar graphs show mean ± SEM of percent Fos enhancement in each structure relative to vehicle controls. *p* values are calculated using Weltch’s *t* test. Corresponding FDR-adjusted q values are provided in Table S1.

Comparisons of taste reactivity scores were analyzed using repeated-measures one-way ANOVA with Greenhouse–Geisser correction, followed by Dunnett’s post hoc tests to assess drug effects (Fig. 1), or by unpaired t-tests with Welch’s correction to assess regional effects (Figs. 2-4). Comparisons of distant Fos mapping data were analyzed using unpaired *t* tests with Welch’s correction (Fig. 5), followed by a Benjamini–Hochberg false discovery rate (FDR) correction across all 22 regions of interest in Fig. 5D. The resulting FDR-adjusted q values alongside the raw *p* values are shown in Table S1. Statistical significance was set at *p* < 0.05; effects with q < 0.05 were considered statistically significant after FDR correction, whereas effects with *p* < 0.05 but q ≥ 0.05 were considered nominally significant. All analyses were performed using Graphpad Prism 10 (GraphPad Software, San Diego, CA, USA), except for the FDR correction, which was calculated using a custom spreadsheet implementing the standard Benjamini–Hochberg procedure.

Further methodological details regarding surgical procedures, drug microinjections, behavioral testing and scoring, and histological analysis are provided in the Supplementary Information.

## RESULTS

### Overview

Microinjections of DAMGO, orexin and muscimol at OTam sites each produced statistically significant enhancements in the number of positive orofacial reactions elicited by the hedonic impact of sucrose taste infusions (Greenhouse–Geisser’s corrected F(1.43, 17.16) = 6.93, *p* = 0.011; ACSF vs. DAMGO: 95% CI [−6.95, −2.75], *p* = 0.0001; ACSF vs. orexin: 95% CI [−8.847, −2.230], *p* = 0.0020; ACSF vs. muscimol: 95% CI [−10.18, −2.746] by Dunnett’s test; Fig. 1A and Fig. S1). Sites producing increased hedonic reactions after DAMGO, orexin, or muscimol were clustered in close anatomical proximity, revealing the OTam subregion to function as a hedonic hotspot able to enhance sweetness hedonic impact. In contrast, none of the drugs enhanced hedonic reactions when microinjected into OTal sites. Instead, DAMGO microinjection at OTal sites slightly suppressed hedonic reactions to sucrose, indicating a suppressive hedonic coldspot (Greenhouse–Geisser’s corrected F(1.87, 20.59) = 1.72, *p* = 0.21; ACSF vs. DAMGO 95% CI [0.51–4.32], *p* = 0.014 by Dunnett’s test; Fig. 1B and Fig. S1). These results indicate a clear anatomical dissociation between OTam and OTal sites in the effects of DAMGO, orexin, and muscimol on hedonic responses to sucrose taste. Following DAMGO, orexin, and muscimol microinjections in OTam, we did not observe hedonic orofacial reactions that were directly elicited as motor reactions prior to the onset of oral taste infusion (i.e., during the period after a drug microinjection but immediately before an oral taste infusion was begun). These reactions should have occurred even in the absence of an oral infusion if the pharmacological microinjection by itself directly induced hedonic reactions without requiring a taste stimulus. However, the opioid, orexin, and muscimol microinjections in OTam did not appear to directly induce orofacial reactions in the absence of a sensory stimulus, despite increasing the number of hedonic orofacial reactions elicited by an oral infusion of a palatable taste. In other words, both the taste infusion and the hotspot drug manipulation appeared to be necessary together in order for ‘liking’ reactions to be enhanced.

Occasionally, rats displayed a few aversive reactions to intraoral infusions of 1% sucrose under the vehicle condition. A repeated-measures one-way ANOVA showed only a trend toward an overall drug effect (Greenhouse–Geisser corrected F(2.34, 28.03) = 3.15, *p* = 0.051). Nevertheless, post hoc Dunnett’s tests revealed that both DAMGO and orexin microinjections into OTam sites significantly reduced these occasional aversive reactions compared with vehicle (ACSF vs. DAMGO: 95% CI [0.681, 5.17], *p* = 0.012; ACSF vs. orexin: 95% CI [0.0674, 4.55], *p* = 0.043), whereas muscimol produced no detectable change (Fig. 1C and Fig. S2). No statistically detectable change in aversive responses was observed for any of the three drugs microinjected into the OTal, although there appeared to be a nominal trend towards increased aversion (Greenhouse–Geisser’s corrected F(1.96, 21.6) = 1.55, *p* = 0.23. Fig. 1D and Fig. S2). These results suggest that opioid or orexin stimulation within the OTam hedonic hotspot can both enhance positive hedonic reactions, and suppress negative ‘disgust’ responses.

Cannula placements were confirmed by fluorescent tracer (Red RetroBeads) and DAPI staining (Fig. 1E). In all 13 rats of the OTam-targeted group, cannula tips were localized within the OT, ranging between AP + 2.2 to +2.7, ML 0.6–1.2, and DV −8.3 to −7.8 mm from bregma. Among the 13 rats of the OTal-targeted group, 12 rats had cannula tips localized within the OT, ranging between AP; +2.2 to +2.7, ML; 1.8 to 3.0, DV; −8.4 to −8.0 mm from bregma; one rat showed a misplaced cannula in the piriform cortex, and its taste reactivity data were excluded from statistical analyses.

### Magnitude of mu-opioid effects on hedonic impact within the OT anteromedial hotspot and anterolateral coldspot

Mu receptor stimulation by DAMGO microinjections within the OTam hotspot nearly doubled the number of hedonic ‘liking’ reactions elicited by sucrose taste (173% of ACSF levels in the same individuals; Fig. 1A). In contrast to these anteromedial enhancements, DAMGO microinjections into the OTal coldspot oppositely decreased hedonic reactions to nearly half of vehicle control levels (61% of ACSF; Fig. 1B). This regional difference between OTam and OTal in the modulation of ‘liking’ reactions to sucrose taste was statistically significant (Welch’s corrected t(20.27) = 6.67, *p* < 0.001; Fig. 2A, B).

Conversely, DAMGO microinjection within the OTam hedonic hotspot further reduced the relatively rare aversive ‘disgust’ responses to sucrose taste to less than half of vehicle control levels (36% of ACSF; Fig. 1C). DAMGO microinjection within the OTal did not significantly alter the number of ‘disgust’ responses. The regional difference in changes of ‘disgust’ reactions to sucrose taste was again statistically significant (Welch’s corrected t(15.28) = 3.00, *p* < 0.01; Fig. 2C, D).

### Magnitude of orexin effects within the OT anteromedial hotspot and anterolateral coldspot

Orexin-A peptide microinjections within the OTam hotspot similarly almost doubled the number of hedonic ‘liking’ reactions elicited by sucrose taste (183% of ACSF; Fig. 1A). In contrast, orexin microinjections within the OTal coldspot did not significantly change the number of hedonic reactions elicited by sucrose (Fig. 1B). This regional difference in enhancement of ‘liking’ reactions to sucrose taste was statistically significant (Welch’s corrected t(19.75) = 3.74, *p* = 0.0013; Fig. 3A-B).

Conversely, aversive ‘disgust’ responses to sucrose taste were suppressed to approximately half of vehicle control levels following orexin microinjections within the OTam hotspot (49% of ACSF; Fig. 1C), whereas orexin microinjection within the OTal coldspot did not significantly affect ‘disgust’ responses. This regional difference in the suppression of ‘disgust’ reactions was statistically significant (Welch’s corrected t(20.84)= 3.55, *p* < 0.01; Fig. 3C, D).

### Magnitude of muscimol effects on hedonic impact within the OT anteromedial hotspot

Muscimol microinjections within the OTam hotspot nearly doubled the number of hedonic ‘liking’ reactions elicited by sucrose taste (198% of ACSF; Fig. 1A). A suppressive coldspot was not detected for muscimol in the OTal, as it did not change the number of hedonic reactions (Fig. 1B). This regional difference between OTam and OTal in the enhancement of ‘liking’ reactions to sucrose taste was statistically significant (Welch’s corrected t(20.75) = 3.87, *p* = 0.0003; Fig. 4A, B).

The number of aversive ‘disgust’ responses to sucrose taste was not significantly affected by muscimol microinjections in either the OTam hotspot or the OTal coldspot (Fig. 1C, D). Muscimol microinjections therefore did not produce a significant regional difference regarding changes of ‘disgust’ reactions to sucrose taste (Welch’s corrected t(19.68) = 1.07, *p* = 0.30; Fig. 4C, D).

### Fos plume and mapping: local and distant anatomical spread of drug impact following DAMGO microinjection in the OT anteromedial hotspot

To assess the primary local activation within the anteromedial OT hotspot and its concomitant co-activation in other regions, we conducted Fos plume and distant mapping analyses for DAMGO microinjections, selected as the representative drug. Fos-expressing cells surrounding the cannula tip were quantified to measure local ‘Fos plumes’ (Fig. 5A and Fig. S3-S4). These Fos plumes typically exhibited a two-layered structure, consisting of inner zones of intense (>200%) increase in Fos cell counts with an average radius of 0.21 mm (volume = 0.039 mm^3^), surrounded by outer zones of moderate (>150%) increases with an average radius of 0.27 mm (volume = 0.083 mm^3^), relative to baseline Fos cell counts measured at equivalent sites in vehicle ACSF-injected control rats (Fig. 5A, B). The averaged diameters of these Fos plumes were used to set the symbol sizes in the remapping of the functional hedonic enhancement sites derived from taste reactivity data (Fig. 5C, remapped from Fig. 2A). The color of each symbol represents the intensity of functional effects induced by DAMGO microinjection at that site, expressed as the within-subject percentage change in hedonic ‘liking’ reactions to sucrose compared to vehicle baseline levels measured in the same rat.

We next assessed distant changes in Fos expression across several mesocorticolimbic structures following DAMGO microinjection within the OTam (Fig. 5D and Fig. S4). Fos cell density were compared with those of control rats that received vehicle ACSF microinjection at equivalent sites. This analysis aimed to test the hypothesis that stimulation of neurons within a particular hedonic hotspot would recruit Fos elevations in other anatomically distant hedonic hotspots, thereby producing concurrent activation across multiple hotspots as a distributed hedonic network mediating ‘liking’ enhancements. DAMGO microinjection into the OTam hotspot induced nominally significant increases in Fos cell density within the caudal VP (212% increase; *p* = 0.022, q = 0.16) and the rostromedial OFC (175% increase; *p* = 0.045, q = 0.198) hedonic hotspots (Fig. 5D and Table S1). We did not detect a significant Fos increase in the NAc rostrodorsal medial shell, another known subcortical hedonic hotspot, although some individual rats exhibited marked Fos elevations. Significant Fos increases were also observed in the rostral ventral tegmental area (*p* = 0.0002, q = 0.004), whereas nominally significant increases were observed in the arcuate nucleus of the hypothalamus (*p* = 0.028, q = 0.152), and medial amygdala (*p* = 0.014, q = 0.154) (Fig. 5D and Table S1).

## DISCUSSION

**The anteromedial OT as a newly identified hedonic hotspot** Our results demonstrate that the anteromedial OT subregion contains a hedonic hotspot capable of enhancing positively valenced orofacial ‘liking’ reactions to sucrose taste following local mu-opioid, orexin, or GABAergic stimulation. Microinjections of DAMGO, orexin-A, or muscimol within the OTam all significantly increased ‘liking’ reactions to sweetness, similarly as reported for the hedonic hotspot in anterodorsal NAc medial shell (and similarly, DAMGO or orexin microinjections also increase hedonic reactions in other hedonic hotspots in posterior VP, anteromedial OFC, and posterior insula) [4, 5, 32]. This finding extends prior evidence that neurons within the OTam contribute to positive behavioral expressions—such as approach [21, 33], investigatory behavior [34], and preferential encoding of stimulus palatability [35]—by identifying this region as a site specifically capable of amplifying hedonic ‘liking’ reactions. More broadly, the present findings are also consistent with the concept that the OT constitutes the tubular striatum [17]. That term recognizes the OT as a striatal-type structure, similar to NAc or neostriatum, which integrates sensory information with motivational state to guide affective and behavioral responses. In this framework, the OTam can be viewed as a functionally specialized subregion within the tubular striatum that can enhance hedonic impact under appropriate conditions.

In contrast, microinjections of the same drugs into the lateral subregion of anterior OT did not enhance ‘liking’ reactions, and DAMGO microinjection there instead suppressed hedonic reactions to sweetness. These findings suggest that OTal may serve as a suppressive ‘hedonic coldspot’ able to reduce positive hedonic impact, similar to coldspots previously reported in the caudal NAc medial shell, rostral VP, caudolateral OFC, and rostral insula [4, 32]. For example, microinjections of mu-, delta-, or kappa-opioid agonists in the caudal coldspot of NAc medial shell all suppress ‘liking’ reactions to sucrose taste [7, 28]. Thus, just as the NAc medial shell exhibits a rostro-caudal gradient of hedonic hotspots and coldspots, the OT may display a corresponding medio-lateral gradient, with the OTam acting as a hedonic hotspot and the OTal as a coldspot that each respectively enhance or suppress ‘liking’ reactions to pleasant sucrose taste.

### The OTam hotspot as a component of a distributed hedonic network

Our histochemical analyses confirmed that DAMGO microinjections in OTam produced local ‘Fos plumes’ (0.21–0.28 mm in radius) of neuronal activation surrounding the microinjection site (Fig. 5). This supports the interpretation that DAMGO acted primarily within the OTam itself and did not spread to adjacent OT subregions or the NAc when enhancing ‘liking’ reactions to sucrose taste. Although Fos expression was most prominent within the dense cell layer of the OTam—suggesting involvement of striatal-like MSNs—the present data do not permit definitive identification of the neuronal subtypes mediating hedonic enhancement. The contribution of other neuronal populations within the OTam remains an important topic for future investigation.

DAMGO microinjection into the OTam also induced distant Fos elevation in other known hedonic hotspots, including the caudal VP and the rostromedial OFC. Notably, the pattern of distant Fos recruitment did not simply mirror anatomical projection density from the OT to target structures: direct anatomical input from the OT to OFC is relatively sparse [36]. These findings suggest that the OTam participates in a distributed hedonic network that coordinates pleasure generation across multiple mesocorticolimbic nodes. This is similar to previous reports that the NAc hotspot in anterodorsal medial shell and the VP hotspot in its poster-olateral subregion are not directly anatomically connected [37] despite functionally recruiting each other into Fos activation during hedonic enhancements [38, 39], suggesting the entire network may function as a unitary whole. Further, several additional mesolimbic structures implicated in reward motivation and appetite, including the rostral VTA, medial amygdala, and arcuate nucleus of the hypothalamus were also activated by DAMGO microinjections into the OTam. One possible interpretation is that hedonic enhancement engages intermediary sites to recruit a selective functional subnetwork within the broader hedonic circuitry. In this view, Fos induction may preferentially reflect regions that are co-activated as part of a coordinated hedonic subnetwork to mediate the amplification of ‘liking’ induced by a hotspot drug microinjection. Further studies are required to delineate the anatomical boundaries of the hedonic hotspot within OTam, or identify the precise circuitry linking the OTam to these downstream structures and to clarify how such network interactions give rise to enhanced hedonic impact.

### Methodological considerations: microinjection and behavioral limits

The present experiments relied on local microinjections of drugs into the OT, together with tracer injections to verify injection sites and assess the physical spread of injected solutions. Although tracer injections indicated that injected solutions could physically diffuse toward adjacent regions outside of OT, physical spread of tracer or drug does not necessarily correspond to functional modulation of neural activity, at least not if neurons with opioid, orexin or GABA receptors have a minimum drug dose threshold that must be exceeded in order to modulate their neuronal function. That is, physical molecules may spread further than functional neuronal modulation if the concentration at furthest spread drops below the dose threshold needed to alter neural function. If so, the diameter of dilute physical spread of tracer molecules may exceed the diameter of neuronal modulation within that zone. We suggest that measured changes in neuronal Fos expression may be our best indicator that a drug microinjection has altered neuronal function: the altered Fos most directly expresses altered neurobiological function induced by drug binding to neurotransmitter receptors. Across behaviorally effective injection sites that enhanced 'liking' reactions, Fos expression consistently formed a compact plume centered within the anteromedial OT, and following anteromedial DAMGO injections local Fos induction was largely confined to the OT subregion itself. We saw little to no change in Fos induction in adjacent portions of either the ventral diagonal band or nucleus accumbens. indicating that local neuronal modulation was primarily restricted to the OT itself. Also, we note that behavioral effects on hedonic taste reactivity were observed only when this Fos plume was centered within the anteromedial subregion of OT, and not when injections were placed outside this region but still in OT. This supports the conclusion that the observed hedonic enhancement effects were driven primarily by local modulation within the OTam.

An additional limitation of the present study is that only a single dose was tested for each pharmacological manipulation, due to the strict limit on the number of microinjections permitted at a single site before local gliosis is induced that reduces drug effectiveness. Although these doses were selected based on prior studies demonstrating robust behavioral effects, dose–response relationships were not systematically examined. Accordingly, it remains possible that different doses could engage additional circuit mechanisms or alter the magnitude and spatial extent of hedonic modulation. Future studies incorporating dose–response analyses will be important for further characterizing the sensitivity and functional organization of hedonic circuits within the OT.

In addition to these methodological considerations related to microinjection and spatial specificity, several behavioral parameters also constrain the interpretation of the present findings. In this study, the sucrose concentration was set at a relatively low level (1%) to prevent potential ceiling effects in ‘liking’ responses to overly sweet sucrose that might obscure further neurobiologically-induced hedonic enhancement. To better characterize OTam enhancement characteristics and to confirm the hedonic suppressive effect of the coldspot in OTal, future taste reactivity studies could employ sucrose solutions of appropriately adjusted concentrations.

Conversely, to test whether neurochemical stimulation of the OTam hedonic hotspot can reduce negatively valenced ‘disgust’ reactions, and whether stimulation of the OTal hedonic coldspot can amplify them, future studies could use bitter quinine solutions [7]. In the present study, only a very few ‘disgust’ gapes or related aversive reactions were observed in response to 1% sucrose, as expected, indicating that a more unpleasant bitter stimulus may be required to evaluate modulation of negatively valenced aversive reactions.

### Neurochemical and circuit mechanisms: opioid, orexin, and GABAergic systems

Cross-drug comparison of injection site effectiveness revealed a moderate degree of spatial concordance across DAMGO, orexin-A, and muscimol, consistent with the presence of a shared functional subdomain within the OTam, while also allowing for drug-specific variability (Table S2). Our data suggest that mu receptor systems in the OTam play a causal role in enhancing ‘liking’ reactions to sweet sucrose. The endogenous opioid system involves β-endorphin-mu receptor, dynorphin-kappa receptor, and enkephalin-delta receptor pairs. Notably, members of all three peptide families can activate each receptor subtype to varying degrees [40]. The OT express mu, kappa, and delta receptors similar to the NAc [41-44]. In addition, MSNs in the OT express preproenkephalin and prodynorphin genes [15]. In the NAc hedonic hotspot, ‘liking’ reactions are enhanced by microinjections of a mu agonist, a delta agonist, or even a kappa agonist [28], and future studies should compare the effects of delta and kappa stimulation in the OTam hotspot. We speculate that activation of the OTam by sensory stimuli may trigger the release of endogenous OT enkephalin and possibly dynorphin under certain conditions, which in turn could amplify hedonic impact [45].

The OTam also shows higher expression of orexin-1 receptor (Hcrtr1) than the lateral OT [46]. This differential expression of orexin receptor may partly underlie the enhanced hedonic ‘liking’ reactions produced by orexin microinjection within the OTam. Local microinjection of an orexin antagonist into the mouse OTam reportedly induces aversive behavioral responses to food associated-odors that are otherwise attractive [47], suggesting that orexin signaling within the OTam contributes to assigning positive valence to odor cues. Orexin microinjections into hedonic hotspots of NAc, VP, OFC, and insula can increase ‘liking’ reactions to sucrose taste similarly to opioid microinjections [5, 25, 48]. Further investigation is needed to determine whether orexin signals in the OTam hotspot enhance ‘liking’ reactions, and might be modulated by changes in physiological states such as those accompanying hunger, satiety, or circadian rhythms.

Muscimol microinjection within the OTam hotspot also enhanced hedonic reactions to sucrose taste, consistent with previous reports of GABA agonist effects within the NAc hedonic hotspot [24]. A major hypothesis of NAc reward function posits that relative inhibition of NAc MSNs mediates reward functions by reducing tonic MSN GABAergic suppression of downstream targets in the VP, VTA, and lateral hypothalamus, thereby releasing those reward-related targets into relative excitation [49-55]. Whether a similar mechanism operates in the OT has remained unknown, but our muscimol results are consistent with the hypothesis that GABAergic inhibition of MSNs in amOT disinhibits downstream targets into relative excitation to cause hedonic enhancement. Among possible targets, both D1- and D2-expressing OT MSNs appear to send axons toward or through the VP, and D1-expressing OT MSNs may form direct synaptic connection with the VTA [12, 56, 57]. This framework also provides a mechanistic context for prior reports implicating D1-expressing neurons in positive motivational and approach-related behaviors within the OT [21, 33, 34]. Further under-standing of the synaptic connectivity and the inhibitory-excitatory dynamics within the OTam will be essential to explain how local GABAergic inhibition by muscimol can ultimately enhance hedonic impact.

### Implications for olfaction, flavor, and food pleasure

As its olfactory label implies, the OT receives dense synaptic inputs from the olfactory bulb and olfactory cortex, which likely convey odor information to the OT. Olfaction plays important roles in flavor perception through the retronasal pathway, an essential contributor to the pleasure of eating [58, 59]. The importance of olfaction in food pleasure is highlighted by clinical findings that patients with olfactory dysfunctions often experience a marked reduction in eating enjoyment [60, 61].

As a speculative hypothesis, the hedonic hotspot identified in the OTam may contribute to the pleasurable impact of flavor sensations, thereby promoting learning and motivation toward particular foods. Supporting this idea, human neuroimaging studies have been shown that subjective pleasantness ratings of odors correlate with activity in the OT [62]. Our identification of a hedonic hotspot within the OTam thus provides new insight into affective neuroscience by revealing a neural substrate that potentially links olfaction, flavor, and the pleasure of eating.

## Supplementary Material

Supplementary material

**Supplementary information** The online version contains supplementary material available at https://doi.org/10.1038/s41386-026-02374-6.

## Data Availability

The data that support the findings of this study are included in the main article and its supplementary information files. Additional data underlying this study are available from the corresponding author upon reasonable request.
