# Supplementary material for: Hedonic hotspot in rat olfactory tubercle: map for mu-opioid, orexin, and muscimol enhancement of sucrose ‘liking’
Source: Neuropsychopharmacology. 2026 Mar 3;51(6):984–96. doi: 10.1038/s41386-026-02374-6 (PMC13084741; doi:10.1038/s41386-026-02374-6)
Supplement: Supplementary file 1 — Supplementary Information [file 41386_2026_2374_MOESM1_ESM.pdf]

Supplementary Information

Hedonic hotspot in rat olfactory tubercle: map for mu-opioid, orexin, and muscimol enhancement of sucrose 'liking'

Koshi Murata, Ph.D.[1,2,3] and Kent C. Berridge, Ph.D.[1]

[1] Department of Psychology, University of Michigan, Ann Arbor, Michigan 48109
[2] Division of Brain Structure and Function, Faculty of Medical Sciences, University of Fukui, Fukui, 910-1193, Japan
[3] Life Science Innovation Center, University of Fukui, Fukui 910-1193, Japan

Correspondence:
Koshi Murata, Ph.D.
University of Fukui
23-3 Matsuoka-Shimoaizuki, Yoshidagun Eiheiji-cho, Fukui, 910-1193, Japan
Tel: +81-776-61-8305
Fax: +81-776-61-8155
Email: kmurata@g.u-fukui.ac.jp

Contents:
Supplementary Materials and Methods
Supplementary Figure S1
Supplementary Figure S2
Supplementary Figure S3
Supplementary Figure S4
Supplementary Table S1
Supplementary Table S2

Supplementary Materials and Methods

Surgical procedures

Rats were anesthetized with isoflurane gas prior to surgery (induction: 4–5%, maintenance: 1–2%) and placed in a stereotaxic apparatus (David Kopf Instruments) with the incisor bar set 5.5 mm below the intra-aural zero. At the onset of surgery, atropine (0.05 mg/kg, i.p.), cefazolin (75 mg/kg, s.c.), and carprofen (5 mg/kg, s.c.) were administered.

Permanent microinjection guide cannulas (9 mm, 26-gauge C315G or 23-gauge C317G; Plastics One) were bilaterally implanted into the OT, targeting either the OTam (n = 13 for the taste reactivity test; n = 12 for Fos mapping) or the OTal (n = 13 for the taste reactivity test). The cannula tips were positioned approximately 2 mm above the dense cell layer of the OT.

For the OTam-targeted group, skull holes were bilaterally drilled at +2.3 mm anterior and ±3.0 mm lateral to bregma, and the guide cannulas were inserted at a 15° angle toward the midline to a depth of 8.0 mm from the skull surface. For the OTal-targeted group, skull holes were bilaterally drilled at +1.8 mm anterior and ±2.8 mm lateral to bregma, and the guide cannulas were vertically inserted (0° angle) to a depth of 8.0 mm. Cannula coordinates were made as bilaterally symmetrical as possible across individuals within each target group. Guide cannulas were anchored to the skull with surgical screws and dental acrylic. Dummy cannulas (33-gauge C315DCS or 30-gauge C317DCS; Plastics One) were inserted and kept in place at all times, except during behavioral testing, to prevent occlusion.

For rats in the taste reactivity group, bilateral intraoral cannulas (polyethylene PE-100 tubing) were implanted during the same surgery to permit oral infusions of sucrose solutions. Oral cannulas entered the oral cavity at the upper cheek pouch lateral to the first maxillary molar, ascended beneath the zygomatic arch, and exited the skin at the dorsal head cap[27]. The cannulas did not disrupt normal feeding behavior.

Postoperatively, rats received carprofen at 24 and 48 h and cefazolin at 24 h after surgery, and were allowed to recover for one week before behavioral testing.

Drug microinjections

Rats were gently cradled by hand on the experimenter's lap during microinjections. Polyethylene PE-20 tubing was connected to microinjection cannulas (33-gauge C315I or 30-gauge C317I, Plastics One) that extended 2 mm beyond the guide cannulas to reach OT targets. Drug or vehicle (artificial cerebrospinal fluid, ACSF) solutions were brought to room temperature (~ 21°C), and inspected to confirm the absence of precipitation before microinjection.

Drugs and vehicle solutions were freshly prepared at the beginning of each test series and stored frozen across consecutive test days. All drugs were dissolved in ACSF and bilaterally microinjected over a 1-min period at a volume of 0.2 μl per side (0.2 μl/min) by syringe pump.

Injectors were left in place for 1 min following microinjection to allow diffusion, after which dummy cannulas were replaced, and rats were immediately placed in the taste reactivity testing chamber.

Four microinjection solutions were tested in each rat: DAMGO, a mu receptor agonist (50 ng/0.2 μl per side); orexin-A peptide (500 pmol/0.2 μl per side); muscimol, a $GABA_A$ receptor agonist (75 ng/0.2 μl per side), and ACSF vehicle alone (0.2 μl per side, vehicle control). Drug doses were selected based on previous studies[24,25,28].

For taste reactivity tests, each rat received bilateral microinjections of only one drug or vehicle per day. The order of drugs and vehicle was counterbalanced among DAMGO, orexin, and ACSF for the first three daily microinjections across rats, with muscimol being tested on the fourth day in all rats.

Taste reactivity tests

The taste reactivity test[2,27,29] was used to measure affective orofacial reactions elicited by intraoral infusion of a sucrose solution (1% w/v). A 1-mL volume of sucrose solution was delivered into the mouth via oral cannula over a 1-min period. Infusions were administered 25 min after microinjections, approximately when peak pharmacological effects could be expected[24,25,28]. To infuse sucrose solution into the mouth, a syringe containing sucrose solution was mounted on a syringe pump and connected via polyethylene tubing (PE-50 attached to a PE-10 delivery nozzle) to the rat's oral cannula. Orofacial and somatic reactions were video-recorded at 30 frame-per-second using a close-up lens and an angled mirror placed underneath the transparent floor to capture ventral views for subsequent video analysis.

Prior to testing, rats were extensively handled to familiarize them with the experimenters. They were then habituated to the test chamber for 25 min on three consecutive days and received a mock microinjection of vehicle ACSF on the third day of habituation.

Taste reactivity video scoring

Hedonic, aversive, and neutral taste reactivity patterns were scored off-line using frame-by-frame video analysis. Hedonic responses were defined as rhythmic midline tongue protrusions, lateral tongue protrusions, and paw licks[1]. Aversive responses were defined as gapes, head shakes, face washes, forelimb flails, and chin rubs. Neutral responses included passive dripping of the solution from the mouth, ordinary grooming, and rhythmic mouth movements.

A time-bin scoring procedure was employed to ensure that taste reactivity components of different relative frequencies still contributed equally to the final totals, so that frequent components such as rhythmic tongue protrusions did not swamp rare but equally informative components, such as lateral tongue protrusions[1]. Specifically, rhythmic mouth movements, passive dripping, and paw licking reactions, which occur in long bouts, were scored in 5-s time bins (e.g., 5 s of continuous paw licking behavior was counted as one bout). Rhythmic midline

tongue protrusions and chin rubs, which occur in shorter bouts, were scored in 2-s bins. Lateral tongue protrusions, gapes, forelimb flails, and head shakes, which typically occur as discrete events, were scored as single occurrences each time they occurred (e.g., one gape equals one occurrence).

Individual totals were calculated separately for hedonic and aversive categories. The hedonic reaction total was quantified as the sum of lateral tongue protrusion, rhythmic tongue protrusion, and paw lick scores. The aversive reaction total was quantified as the sum of gape, head shake, face wash, forelimb flail, and chin rub scores.

Histology for cannula placement and mapping of behavioral effects

Following behavioral testing, rats received microinjection of a fluorescent tracer (Red RetroBeads; 0.2 μL per side) through the microinjection cannulas to allow subsequent anatomical mapping of injection sites. Rats were then deeply anesthetized with an overdose of sodium pentobarbital, and perfused transcardially with 0.1M sodium phosphate buffer (NaPB), followed by 4% paraformaldehyde. Brains were removed, post-fixed in 4% paraformaldehyde for 1 day, and then immersed in 25% sucrose (in 0.1 M NaPB) for 1-2 days. Brains were frozen, coronally sectioned at 50 μm on a freezing microtome, mounted, air-dried, and coverslipped using DAPI-containing mounting medium (ProLong Gold Antifade Mountant with DAPI). Bilateral microinjection sites for each rat were assessed by fluorescence microscope, and plotted onto corresponding coronal maps adapted from a rat brain atlas[30]. These plots were used to extrapolate the locations of individual injection sites onto composite group maps of the OT to identify functional hedonic hotspots and coldspots.

Group maps of the behavioral effects of OT microinjections were constructed in the coronal plane to display all injection sites on the same map, revealing functional differences across the mediolateral extents of the anterior OT. Functional effects on hedonic reactions were visualized using color-coding to represent the percentage change in affective behaviors for each rat, calculated as the number of hedonic or aversive reactions during drug conditions divided by the number of corresponding reactions elicited by sucrose under the vehicle ACSF condition (Figs. 2-4). To avoid division by zero for rats that showed no hedonic or aversive reactions under ACSF, we added +1 to the number of reactions in all microinjection conditions (ACSF, DAMGO, orexin, and muscimol) for those rats before calculating percentages. Map symbols indicate the putative tip position of the injection cannulas, as verified by fluorescent tracer labeling.

Histological Fos analysis following DAMGO microinjection in the OTam hotspot

Fos analysis was conducted in a separate group of rats from those used in taste reactivity tests to reveal the anatomical spread of neural activation caused by drug microinjections. Distant Fos effects were also examined in the same rats to determine whether DAMGO in the OTam

recruited neuronal activity in other mesocorticolimbic structures, including other hedonic hotspots such as in orbitofrontal cortex, ventral pallidum, etc. Rats in the Fos analysis group received DAMGO or vehicle ACSF microinjection in the OTam under conditions identical to those of the behavioral group on their first test day. Cannula placements in the Fos group corresponded to those in the taste reactivity test group.

Immunohistochemistry

Eighty-five minutes after microinjection, rats were deeply anesthetized with a lethal dose of sodium pentobarbital (150-200 mg/kg) and transcardially perfused with 0.1M NaPB followed by 4% paraformaldehyde. Brains were removed, post-fixed in 4% paraformaldehyde for 1 day, and transferred to 25% sucrose solution (in 0.1 M NaPB) for 1-2 days. Coronal sections (25 μm) were cut on a cryostat (Leica) and processed for Fos immunohistochemistry.

Sections were rinsed three times for 10 min in 0.1 M NaPB, blocked in 5% normal donkey serum / 0.2% Triton-X in PBS for 60 min, and incubated overnight with a polyclonal rabbit anti-c-Fos primary antibody (1:1000; MERCK/Sigma-Aldrich ABE457). After three 10-min rinses in 0.1M NaPB, sections were incubated for 2-h with a Cy3-conjugated donkey anti-rabbit secondary antibody (1:500; Jackson ImmunoResearch). Sections were rinsed again, mounted, air-dried, and coverslipped with a DAPI-containing mounting medium (ProLong Gold Antifade Mountant with DAPI). Fos immunoreactivity was imaged using a fluorescence slide scanner (VS200, Evident Scientific), and images of whole-brain coronal sections were captured at 20x magnification using Olyvia software.

Local Fos Plume Analysis

Fos plumes were mapped at 20x magnification by counting Fos-positive cells within consecutive 50 μm x 50 μm tissue blocks along seven radial arms emanating from the center of the microinjection site (45°, 90°, 135°, 180°, 225°, 270°, and 315°, Fig. S3). Counting proceeded outward along each arm until at least two consecutive blocks contained no Fos-positive cells. Percent increases in DAMGO-induced Fos expression were calculated relative to baseline levels measured at the same sites in vehicle-injected control rats (Fig. 5A). Fos plumes were defined as zones of intense elevation (>200%) or moderate elevation (>150%), and radii were averaged across the seven arms to determine plume size. The averaged plume radii were used to scale symbol sizes in functional maps of hedonic enhancement sites (Fig. 5C). Cannula tip locations were plotted onto corresponding atlas maps to construct OTam functional localization figures (Fig. 5C, remapped from Fig. 2A).

Distant Fos Mapping Analysis

Functional activation of circuitry recruited by DAMGO microinjection into the OTam hotspot immediately prior to euthanasia was assessed by quantifying Fos cell density at distant sites

across multiple structures: OFC, prelimbic cortex, infralimbic cortex, insula, anterior cingulate cortex (ACC), NAc medial shell and core, VP, central amygdala (CeA), basolateral amygdala (BLA), medial amygdala (MeA), lateral hypothalamus (LH), perifornical area of hypothalamus (PFA), arcuate nucleus of hypothalamus, VTA, and paraventricular thalamus.

Within each subregion, Fos-positive cells were counted in two to six sample boxes (200 µm x 200 µm), placed equidistantly within the structure, and approximately matched across rats, guided by a template derived from a corresponding brain atlas to ensure consistent placement[31]. The number of sample boxes was adjusted for each structure to capture an average 6–15 Fos-positive cells in vehicle-injected control rats. Counts across sample boxes were summed to determine total Fos cell density for each subregion or structure.

Supplementary Figure S1

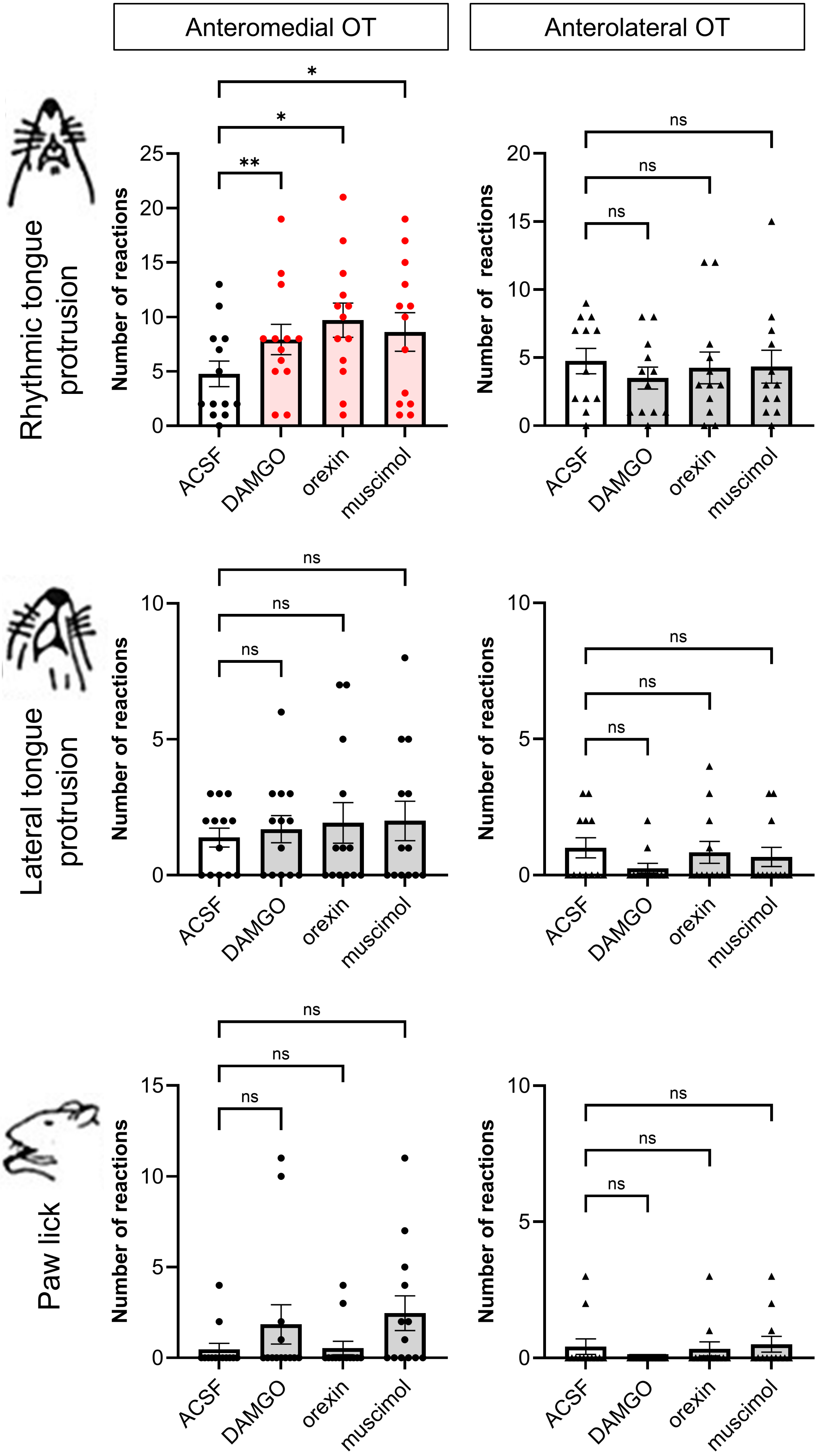

Supplementary Figure S1. Breakdown of hedonic ‘liking’ reactions.

Fig. S1 decomposes the total hedonic ‘liking’ reaction scores shown in Fig. 1A,B into their constituent affective facial expressions. Left panels depict rats receiving drug microinjections into the anteromedial OT, and right panels depict injections into the anterolateral OT. The three rows show the positive ‘liking’ components scored during sucrose tasting: top, rhythmic tongue protrusions; middle, lateral tongue protrusions; bottom, paw licks. For each drug condition, values represent the mean number of reactions elicited during the taste reactivity test. Summing these three affective components yields the total ‘liking’ scores presented in Fig. 1A,B. $^*p < 0.05$, ns not significant. n = 13 rats for anteromedial OT; n = 12 rats for anterolateral OT.

Supplementary Figure S2

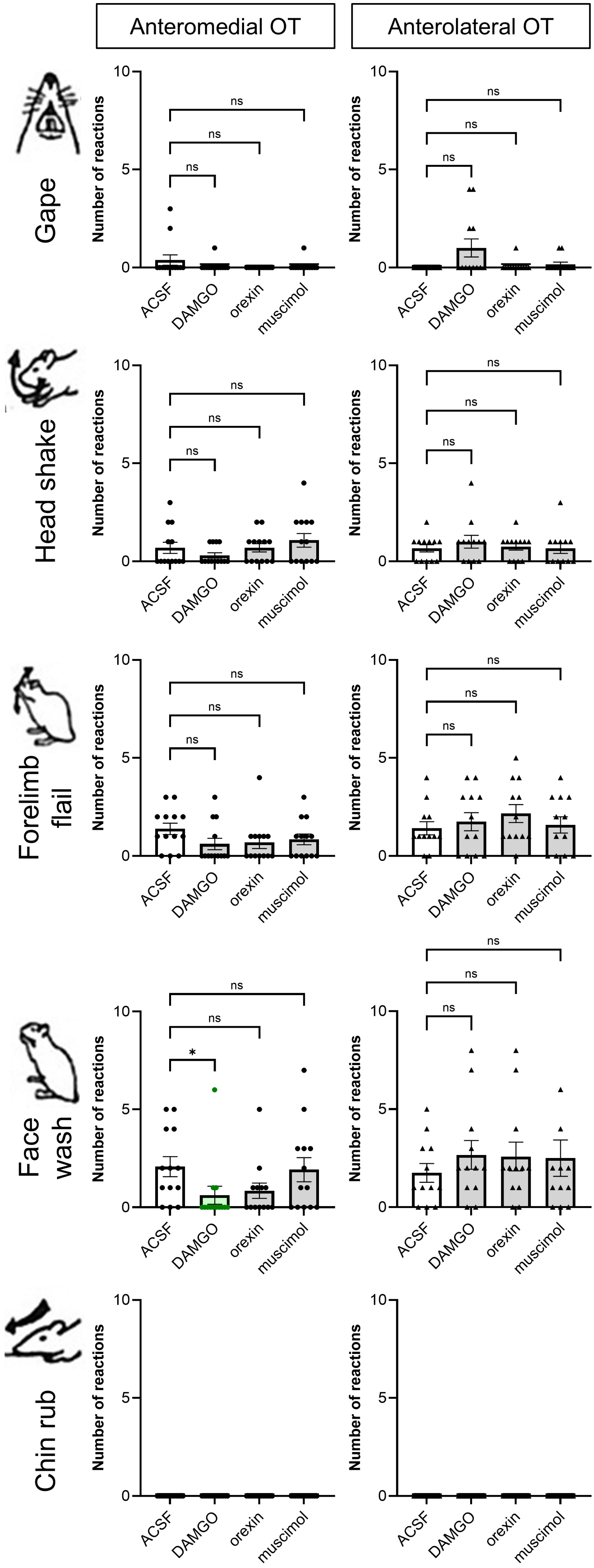

Supplementary Figure S2. Breakdown of aversive ‘disgust’ reactions.

Fig. S2 decomposes the total aversive ‘disgust’ reaction scores shown in Fig. 1C,D into their constituent negative affective facial and somatic expressions. Left panels depict rats receiving drug microinjections into the anteromedial OT, and right panels depict injections into the anterolateral OT. The five rows illustrate the aversive ‘disgust’ components scored during sucrose tasting under drug conditions: top, gapes; second, head shakes; third, forelimb flails; fourth, face washes; bottom, chin rubs. For each drug condition, values represent the mean number of reactions elicited during the taste evaluation period. Summing these five aversive components yields the total ‘disgust’ scores presented in Fig. 1C,D. $^*p < 0.05$, ns not significant. $n = 13$ rats for anteromedial OT; $n = 12$ rats for anterolateral OT.

Supplementary Figure S3

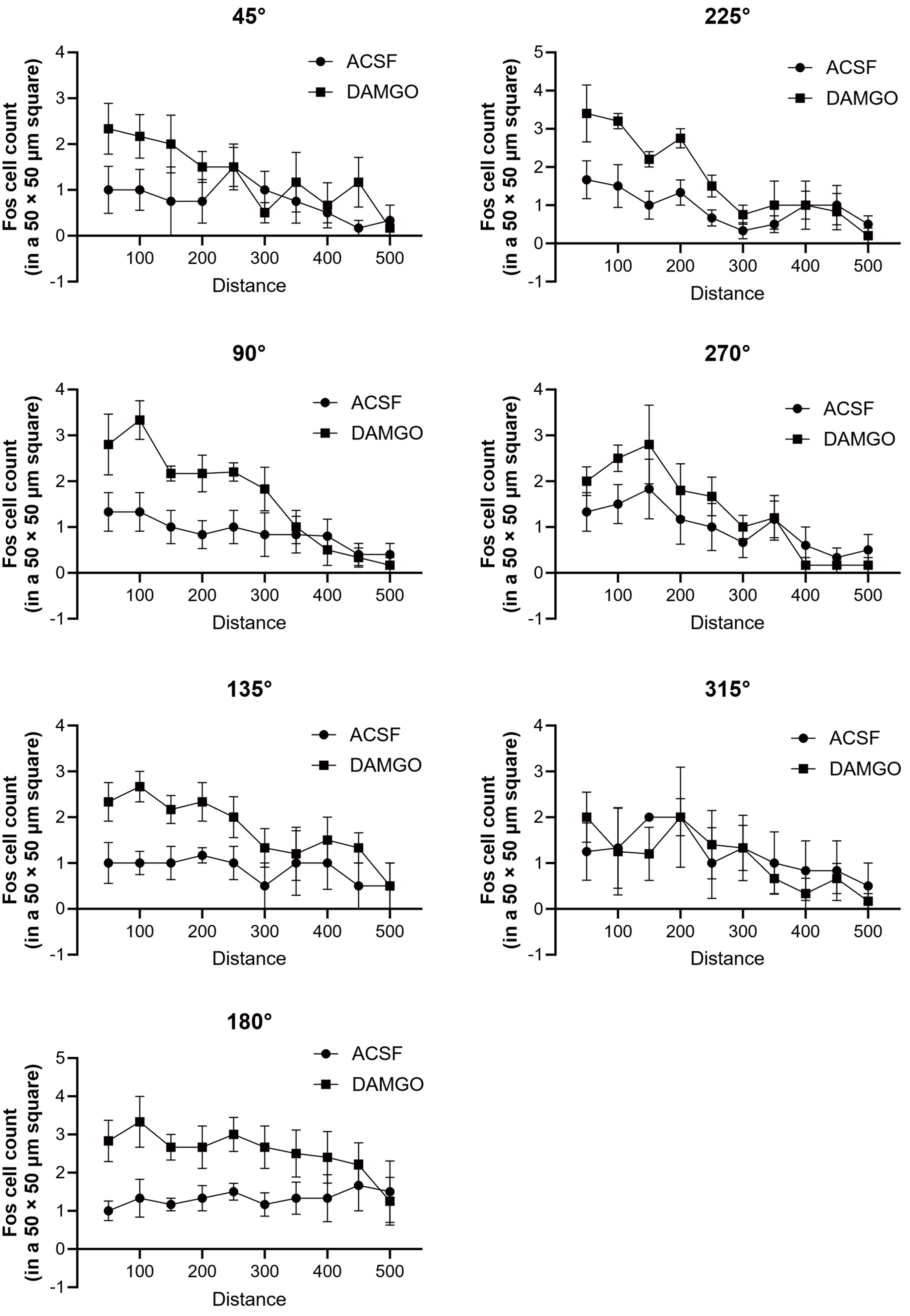

Supplementary Figure S3. Arm-specific Fos cell counts within the local Fos plume. Quantification of Fos cells within the local Fos plume, analyzed separately for each anatomical arm extending radially from the microinjection center. Arms were defined at 45° increments (45°, 90°, 135°, 180°, 225°, 270°, and 315°) relative to the injection center. Each arm was constructed by consecutively arranging square sampling boxes (50 µm × 50 µm) along the specified direction. Fos cells were counted within each box, corresponding to the local Fos plume illustrated in Fig. 5A. In the line plots, Fos cell count values for individual boxes are plotted as a function of distance from the injection center, illustrating spatial gradients of Fos induction along each plume arm.

Supplementary Figure S4

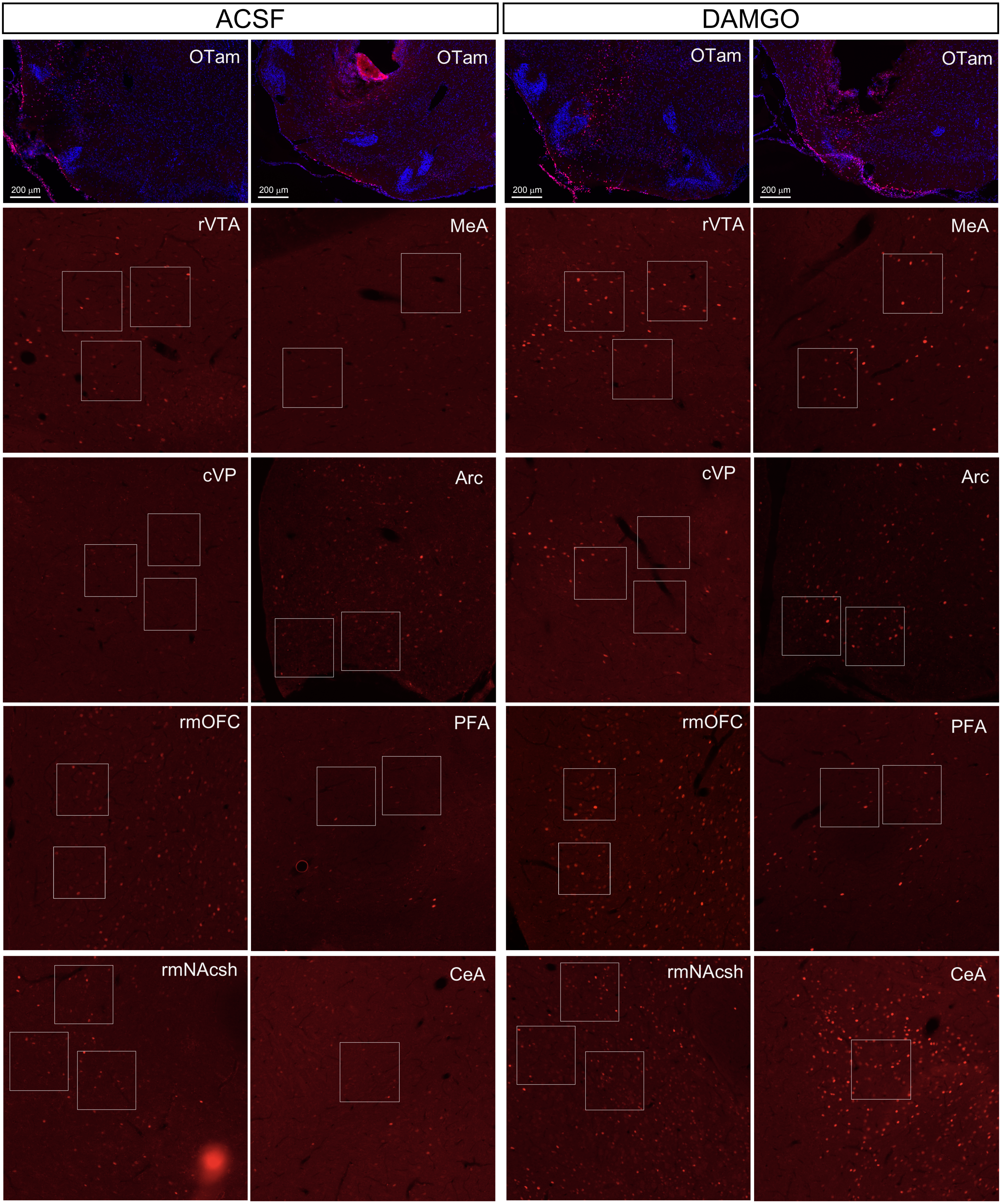

Supplementary Figure S4. Representative Fos photomicrographs from the OT and related regions.

Representative photomicrographs showing Fos expression in selected brain regions. Scale bars are included in OTam images. Boxed areas indicate regions of interest (200 μm × 200 μm). In the OTam, Fos immunoreactivity is shown in red with DAPI counterstaining in blue, whereas images from all other regions display Fos immunoreactivity alone (red). OTam, anteromedial olfactory tubercle; rVTA, rostral ventral tegmental area; MeA, medial amygdala; cVP, caudal ventral pallidum; Arc, arcuate nucleus; rmOFC, rostromedial orbitofrontal cortex; PFA, perifornical area; rmNAcsh, rostromedial nucleus accumbens shell; CeA, central amygdala.

Supplementary Table S1. Statistical metrics for distant Fos mapping analysis.

Region	Mean Fos cell density (/mm^2) ACSF	Mean Fos cell density (/mm^2) DAMGO	ΔACSF (%)	t value	Degree of freedom	p value	FDR-adjusted q value
Rostral VTA	69	138	201	5.56	10	0.0002	0.004
Medial Amygdala	81	123	151	2.97	10	0.014	0.154
Caudal VP	19	40	212	2.70	10	0.022	0.164
Arcuate Nucleus	56	117	207	2.57	10	0.028	0.152
Rostromedial OFC	134	235	175	2.29	10	0.045	0.198
Perifornical Area	50	85	169	1.81	10	0.100	0.368
NAc rostrodorsal medial shell	47	116	249	1.65	10	0.129	0.406
Central Amygdala	107	234	219	1.27	10	0.234	0.576
Rostral ACC	89	148	167	1.18	10	0.265	0.576
PVT	221	285	129	1.14	10	0.279	0.576
Caudal VTA	28	22	78	1.06	10	0.316	0.576
Caudolateral OFC	100	142	142	1.01	10	0.337	0.576
Basolateral Amygdala	129	221	171	1.00	10	0.340	0.576
NAc core	42	82	195	0.94	10	0.371	0.582
Rostral insula	37	54	146	0.82	10	0.434	0.592
Mid ACC	99	136	137	0.77	10	0.461	0.592
NAc caudomedial shell	42	56	133	0.73	10	0.481	0.592
Infralimbic	88	120	137	0.73	10	0.484	0.592
Prelimbic	94	122	130	0.63	10	0.542	0.628
Caudal insula	58	80	138	0.52	10	0.612	0.673
Lateral Hypothalamus	54	61	113	0.41	10	0.694	0.727
Rostral VP	43	46	107	0.22	10	0.832	0.832

VTA, ventral tegmental area; VP, ventral pallidum; OFC, orbitofrontal cortex; NAc, nucleus accumbens; ACC, anterior cingulate cortex; PVT, paraventricular thalamus.

Supplementary Table S2. Spatial effectiveness of injection sites across pharmacological manipulations.

Rat ID	Cannula tip (Left OT)			Cannula tip (Right OT)			% Vehicle Change		
	AP	ML	DV	AP	ML	DV	DAMGO	Orexin	Muscimol
#1	2.7	0.8	-7.9	2.7	1.1	-8	138	163	188
#2	2.2	1.1	-8.1	2.2	1.2	-8.2	800	1400	300
#3	2.2	0.8	-8.1	2.2	1.1	-8.2	200	200	700
#4	2.2	0.8	-8	2.2	0.8	-8	200	180	240
#5	2.2	0.7	-7.9	2.2	0.6	-8	136	136	145
#6	2.7	1	-8.1	2.7	1.1	-8.2	131	94	163
#7	2.2	0.9	-8	2.2	1.1	-8.1	200	150	170
#8	2.2	1.2	-8.3	2.2	1.1	-8	188	138	275
#9	2.2	0.9	-8.3	2.2	0.7	-8.3	109	191	136
#10	2.2	0.6	-7.8	2.2	1	-8.2	260	240	240
#11	2.2	1.1	-8	2.2	0.7	-8	150	450	25
#12	2.2	0.8	-7.9	2.2	0.9	-8.1	200	167	633
#13	2.2	0.6	-7.9	2.2	0.7	-7.8	275	225	150

Each of the 13 injection sites is listed with stereotaxic coordinates (mm from bregma) and its effectiveness category for DAMGO, orexin-A, and muscimol. Effectiveness categories were defined based on percent change in hedonic reactions relative to vehicle and are indicated by color coding (red, orange, yellow, gray, light blue, and blue), matching the color scheme used in Figs. 2-4. This table provides a descriptive summary of cross-drug consistency in spatial effectiveness, which was further quantified using Kendall's coefficient of concordance, revealing a moderate level of agreement ($W = 0.60$).
